# Rheumatoid arthritis following PEG-interferon-alfa-2a plus ribavirin treatment for chronic hepatitis C: a case report and review of the literature

**DOI:** 10.1186/1756-0500-6-437

**Published:** 2013-10-30

**Authors:** Bruno Cacopardo, Francesco Benanti, Marilia Rita Pinzone, Giuseppe Nunnari

**Affiliations:** 1 Department of Clinical and Molecular Biomedicine, Division of Infectious Diseases, University of Catania, Via Palermo 636, 95125, ARNAS Garibaldi Nesima, Catania, Italy

## Abstract

**Background:**

The combination of Pegylated Interferon-alpha (PEG-IFN-α) and ribavirin is the current standard of care for the treatment of HCV infection. Unfortunately, IFN-α may lead to the induction or exacerbation of autoimmune diseases, such as psoriasis, thyroiditis, systemic lupus erythematosus and, rarely, rheumatoid arthritis (RA).

**Case presentation:**

We report the case of a man affected with chronic hepatitis C (CHC) due to HCV genotype 3a infection, who developed RA after a complete course of PEG-IFN-α and ribavirin. Nine weeks after cessation of antiviral treatment, the patient developed symmetrical polyarthritis, with pain and edema in the wrists, knees, shoulders and metacarpophalangeal joints; magnetic resonance imaging detected initial bone erosions with juxta-articular osteopenia in wrist, knee and hand joints. Anti-cyclic citrullinated peptide (anti-CCP) antibodies were positive.

**Conclusions:**

Autoimmune diseases, including RA, may occur when treating chronic hepatitis C with PEG-IFN-α and ribavirin; therefore, a close surveillance for the occurrence of autoimmune phenomena should be suggested in the setting of HCV management.

## Background

Hepatitis C Virus (HCV) is the most common cause of cirrhosis and hepatocellular carcinoma (HCC) in Western countries, both in the immunocompetent and in the immunodeficient host [1,2].

The combination of Pegylated Interferon-alpha (PEG-IFN-α) and ribavirin is the current standard of care to treat HCV infection [3]; unfortunately, this therapy is accompanied by a wide variety of possible side effects, which may lead to early or late treatment discontinuation [4]. Ongoing studies have focused on new therapeutic molecules and strategies, in order to find IFN-free regimens, with a better tolerability profile [5,6].

Autoimmune phenomena have been frequently reported in patients with chronic HCV infection receiving IFN-α treatment [7-9]. The spectrum of autoimmune side effects ranges from asymptomatic appearance of serum autoantibodies up to development of overt autoimmune diseases [9,10]. Although rheumatoid arthritis (RA) is one of the most common autoimmune diseases, the development of RA after IFN-α plus ribavirin has rarely been reported [11-14] (Table 1). We report the case of a 53-year-old man with Chronic Hepatitis C (CHC), who developed RA after a complete course of PEG-IFN-α and ribavirin therapy.

**Table 1:** Previous literature reports of IFN-α-induced rheumatoid arthritis in patients with CHC

	**Age**	**Sex**	**Treatment**	**Onset**	**Biochemical abnormalities**	**Treatment and outcome**
Sood *et al.*[12]	47	W	Recombinant IFN-α-2b + RIB	20 weeks after starting antiviral therapy	ESR↑, ANA-, RF+	NSAIDs
						No interruption of antiviral therapy
Ionescu *et al.*[13]	40	W	PEG-IFN-α-2b + RIB	45 weeks after starting antiviral therapy	ESR↑, RF+, Fibrinogen ↑, IgG ↑	Analgesics and NSAIDs
			PEG-IFN-α-2a + RIB	10 months after re-treatment for relapsing CHC	HLA B27- HLA DR3/4+	Regression after cessation of antivirals
Yang *et al.*[14]	54	M	PEG-IFN-α-2a + RIB	18 weeks after starting antiviral therapy	ESR↑, ANA-, RF-, Anti-CCP Ab+, CRP ↑,	Ibuprofen, celecoxib and tramadol, then switch to hydroxychloroquine and sulfasalazine
Izumi *et al.*[11]	48	M	PEG-IFN-α + RIB	2 months after cessation of antivirals	ANA+, RF-, ESR↑, Anti-CCP Ab+, BAFF↑	Methotrexate and sulfasalazine

Anti-CCP Ab: anti-cyclic citrullinated protein antibody; ANA: antinuclear antibody; BAFF: B-lymphocyte activating factor; CHC: chronic Hepatitis C; CRP: C-reactive protein; ESR: Erythrocyte Sedimentation Rate; M: man; NSAIDs: Non-steroidal anti-inflammatory drugs; PEG-IFN: Pegylated interferon; RF: rheumatoid factor; RIB: ribavirin; W: woman; +: positive; ↑: increased.

## Case presentation

A 53-year-old man, working as a male nurse in a local hospital, was diagnosed with HCV infection after detection of abnormal liver function tests in 2010. His past medical history was unremarkable. He denied intravenous drug abuse or history of blood transfusions. In January 2011 he presented to the Outpatient Infectious Diseases clinic for evaluation: he was in good clinical condition and did not complain at all of articular or muscular pain; liver was palpable 3 cm below the right costal margin. No splenomegaly was present. His Body Mass Index was 27. HCV RNA was 660,000 IU/mL (TaqMan Real Time PCR); HCV genotype was 3a (INNO-LiPA HCV; Innogenetics, Ghent, Belgium). Liver biopsy showed a chronic active hepatitis, with Metavir A2 necroinflammatory grading and F2 fibrosis. FibroScan value was 6.1 kPa. Alanine aminotransferase (ALT) was over two times the upper limit of normal; thyroid hormones were normal as well as serum autoantibodies. After a psychiatric exam, which was negative for depressive disorders, the patient was considered eligible for antiviral treatment.

Table 2 illustrates in detail biochemical and virological parameters prior to antiviral treatment initiation.

**Table 2:** Biochemical, virological, histological and immunological parameters before and after a 24-week course of PEG-IFN-α-2a and ribavirin in a CHC patient who developed post-treatment RA

	**Pretreatment**	**Post-treatment**
AST (IU/mL)	66	25
ALT (IU/mL)	84	19
HCV RNA (IU/mL)	660,000	negative
Liver histology	A2/F2	A1/F1
METAVIR grading/staging		
Erythrocyte sedimentation rate (1st hour)	11	76
C-reactive protein (mg/dL)	0.35	2.21
Rheumatoid Factor	negative	positive
Anti-CCP Antibodies (IU/ml)	negative	860
Antinuclear Antibodies	negative	1/320
Anti-dsDNA Antibodies	negative	negative
Cryoglobulin	negative	negative

ALT: alanine aminotransferase; AST: aspartate aminotransferase; anti-CCP: anti-cyclic citrullinated protein; CHC: cronic hepatitis C; dsDNA: double-stranded DNA; RA: rheumatoid arthritis.

In March 2011 antiviral therapy was started with PEG-IFN-α-2a (180 mcg per week subcutaneously) and ribavirin (1000 mg per day orally). This therapy was prolonged for as long as 24 weeks. HCV-RNA became negative by the fourth week and persisted undetectable up to treatment completion. Similarly, alanine aminotransferase (ALT) persistently normalized within the first 3 weeks of treatment. Treatment was well tolerated, with the exception of flu-like symptoms, easily controlled by paracetamol, and a mild thrombocytopenia (platelet count nadir of 88,000/μl). In September 2011, the patient started the post-treatment follow up which confirmed the persistence of normal ALT and negative HCV RNA throughout 48 weeks up to September 2012. In September 2012, a new liver biopsy showed a mild reduction of Metavir grading and staging (A1 and F1, respectively).

Nine weeks after the cessation of PEG-IFN and ribavirin (November 2011), the patient developed symmetrical polyarthritis, with pain and edema in the wrists, knees, shoulders and metacarpophalangeal joints, associated with prolonged morning stiffness. Distal interphalangeal joints were spared. Hand X-ray showed no remarkable findings, but magnetic resonance imaging (MRI) detected bone erosions with juxta-articular osteopenia in wrist, knee and hand joints. Laboratory exams revealed a white blood cell count (WBC) of 7,200 cells/μl, haemoglobin level was 12.8 g/dL, creatinine was 0.9 mg/dL. Post-treatment biochemical, virological and immunological data are summarized in Table 2.

A diagnosis of rheumatoid arthritis (RA) was made on the basis of clinical features, MRI evidence of juxta-articular bone erosions, Rheumatoid Factor (RF) and anti-cyclic citrullinated peptide (anti-CCP) antibody positivity.

The patient was initially treated with prednisone 25 mg/day orally and Nonsteroidal Anti-inflammatory Drugs (NSAIDs) for as long as two months; in April 2012 he was switched to methotrexate (6 mg/week) and sulfasalazine (2 grams/day), due to the persistence of joint pain, obtaining a quick favorable response to treatment.

## Conclusions

HCV-related inflammatory arthritis has been described as falling into two subsets. One subset is associated with mixed cryoglobulinemia and is usually monoarticular [15]. The other subset is a symmetrical RA-like polyarthritis lacking juxta-articular erosions and rheumatoid nodules [15]. Differently from patients affected with overt RA, patients with HCV-related arthritis also lack anti-CCP titers [11,15]. Another relevant characteristic of HCV-related arthritis resides in the fact that it frequently improves after treatment with IFN-α, even without achieving a complete virological response, possibly as a consequence of decreased viral load [15].

Standard combination therapy of PEG-IFN-α and ribavirin in CHC subjects is associated with a sustained virological response (SVR) in more than 50% of patients [16]. A number of predisposing conditions [17], including the presence of a clear-cut liver cirrhosis, could negatively affect the outcome of such therapy [18]. In addition, antiviral treatment is accompanied by a wide variety of possible side effects. The most frequent IFN side effects, which have been described not only in CHC, but also in acute hepatitis C [19] and chronic hepatitis B [20] are flu-like symptoms, hematologic abnormalities, such as leukopenia and thrombocytopenia, and psychiatric changes, such as irritability and depression. Moreover, immunomodulatory effects of IFN may lead to the induction or exacerbation of autoimmune diseases, including psoriasis, thyroiditis, systemic lupus erythematosus and rarely RA [7,11-14,21-23]. IFN-α determines a shift of T-lymphocyte responses towards a T-helper (Th)-1 profile, inhibiting the production of Interleukin (IL)-10 and stimulating the release of tumor necrosis factor (TNF)-α and IL-12. In addition, IFN-α has been found to induce the production of B-lymphocyte activating factor (BAFF) both in mice with lupus erythematosus [23] and patients with multiple sclerosis [24]. Considering that BAFF levels have been shown to correlate with autoantibody levels and synovitis in a subset of patients with early RA [25], it may be hypothesized that BAFF induction, occurring during treatment with IFN-α, may favor the development of RA in susceptible individuals. Indeed, Izumi et al. [11] found that BAFF titers were markedly higher after starting antiviral treatment with IFN plus ribavirin in comparison with pretreatment values.

Only 4 cases of RA associated with recombinant or Pegylated-IFN-α treatment for CHC have been previously reported in the literature [11-14] (Table 1). In the aforementioned case report of Izumi et al. [11], RA developed three months after cessation of antiviral treatment, whereas in the other reports [12-14] RA occurred during treatment, with an interval ranging from 10 to 42 weeks of therapy. PEG-IFN-α was administered in 3 of 4 cases, whereas in the remaining case recombinant IFN-α-2b was used [12]. Anti-CCP antibodies tested positive only in two of four case reports [11,14]. Of interest, Ionescu et al. [13] presented the case of a 45-year-old woman who had developed RA during treatment with PEG-IFN-α-2b and ribavirin, whose retreatment with PEG-IFN α-2a and ribavirin for relapsing CHC caused the reappearance of RA. In our case, both the detection of anti-CCP antibodies and the presence of erosive disease allowed to distinguish RA from typical HCV-related arthritis. In addition, our patient developed arthritis after cessation of PEG-IFN-α plus ribavirin treatment, when HCV RNA was no longer detectable. Therefore, a diagnosis of IFN-induced RA was more likely than one of HCV-associated arthritis.

In conclusion, we report a case of RA occurring after a successful course of PEG-IFN-α plus ribavirin for the treatment of CHC. The present case suggests that biological agents, affecting the cytokine network, may work as triggering factors for the development of RA. Before treating CHC patients with PEG-IFN-α and ribavirin, screening for anti-CCP levels may be considered; in addition, a close surveillance for the occurrence of autoimmune phenomena during and after treatment should be worthy, for early diagnosis and adequate clinical management.

## Consent

Written informed consent was obtained from the patient for publication of this case report. A copy of the written consent is available for review by the Editor-in-Chief of this journal.

## Competing interests

The authors declare that they have no competing interests.

## Authors’ contributions

BC and FB provided study material. BC and MRP wrote the paper. GN contributed to literature research and revised the paper. All authors read and approved the manuscript to be published.
